# Encountering existential loneliness among older people: perspectives of health care professionals

**DOI:** 10.1080/17482631.2018.1474673

**Published:** 2018-06-05

**Authors:** Malin Sundström, Anna-Karin Edberg, Margareta Rämgård, Kerstin Blomqvist

**Affiliations:** a Research Platform for Collaboration for Health, Faculty of Health Science, Kristianstad University, Kristianstad, SWEDEN; b Faculty of Health and Society, Malmö University, Malmö, SWEDEN

**Keywords:** Existential loneliness, older people, health care professionals, qualitative study, focus group, encounter, life world

## Abstract

**Purpose**: Existential loneliness is part of being human that is little understood in health care, but, to provide good care to their older patients, professionals need to be able to meet their existential concerns. The aim of this study was to explore health care professionals’ experiences of their encounters with older people they perceive to experience existential loneliness. **Method**: We conducted 11 focus groups with 61 health professionals working in home care, nursing home care, palliative care, primary care, hospital care, or pre-hospital care. Our deductive–inductive analytical approach used a theoretical framework based on the work of Emmy van Deurzen in the deductive phase and an interpretative approach in the inductive phase. **Results**: The results show that professionals perceived existential loneliness to appear in various forms associated with barriers in their encounters, such as the older people’s bodily limitations, demands and needs perceived as insatiable, personal shield of privacy, or fear and difficulty in encountering existential issues. **Conclusion**: Encountering existential loneliness affected the professionals and their feelings in various ways, but they generally found the experience both challenging and meaningful.

## Introduction

To avoid unnecessary suffering in older people, health care professionals in all professions and care contexts need to meet these people’s existential concerns. Although previous research shows that nursing staff are willing to pay attention to existential issues (Strang, Strang, & Ternestedt, 2002), more recent research shows that it is difficult and challenging to find time and space for this in everyday elderly care (Beck, 2013; Norell Pejner, 2013; Sundler, Eide, Van Dulmen, & Holmström, 2016). This can lead nursing staff to feel compelled to focus more on practical tasks than on relations with the older people and their relatives (Beck, 2013). In turn, when care is focused more on tasks than on the relational aspects that allow a good encounter, older people often feel alienated and lonely (Svanström, Sundler, Berglund, & Westin, 2013). The concept of *loneliness* is ambiguous and includes both the objective dimension of being alone and the subjective dimension of feeling lonely despite having people around (Dahlberg, 2007). This study has its starting point in existential aspects of loneliness, *existential loneliness* in a caring context, and how professionals experienced existential loneliness in the encounter with older people.

Loneliness has been described extensively in relation to death, guilt, and other existential aspects of being human, often by and with reference to philosophers such as Frankl, Heidegger, and Yalom. Although loneliness has been described as a part of being human (Yalom, 1980) and a part of the human predicament (Tillich, 1963) in terms such as *aloneness, solitude*, and *isolation*, no clear consensus has been reached on a definition of the concept in general (Karnick, 2005) or of existential loneliness in particular (Ettema, Derksen, & van Leeuwen, 2010). Loneliness also has different aspects and meanings. Dahlberg (2007) described loneliness as restful and creative, and Tillich (1963) distinguished loneliness, *the pain of being alone*, from solitude, *the glory of being alone*, and emphasized the greater significance of loneliness to the health and care of a person suffering and in need of care. It is therefore reasonable to believe that health care professionals have seen both loneliness and existential loneliness in the older people in their care.

Existential loneliness is related to feeling disconnected from the world, lost without a purpose, and adrift in life. Existential loneliness can also arise when people lack previous experience of their situation or in times of uncertainty such as during an illness. Jaspers (1994) defined ʽlimit situationsʼ (i.e., death, suffering, struggling, faith, and guilt) as closely connected to life, and therefore inevitable and unescapable, whereas Tillich (1963) defined guilt and death as two forms of loneliness that cannot be covered up or escaped. According to Applebaum (1978), the full impact of existential loneliness is often felt during the contemplative realization of one’s aloneness in the universe, and responses can vary from fright to excitement and acceptance of reality and one’s autonomy. Ettema et al. (2010) described three dimensions of existential loneliness as (1) a condition, (2) an experience, and (3) a process of inner growth, indicating its positive as well as negative aspects, which concern aspects of being with others in fellowship and connectedness, and of being without others.

For most people, ageing brings losses of important aspects of life such as family members, friends, abilities, and physical functions. The impact of these losses and how they are managed seem to be associated with feelings of loneliness (Kirkevold, Moyle, Wilkinson, Meyer, & Hauge, 2013). Unlike separation anxiety, loneliness arises when a loss has occurred, rather than when it is feared. Although the two feelings can occur simultaneously, they should not be confused with each other (Applebaum, 1978). Harris (2015) described existential losses during the course of a life-limiting illness (motor neuron disease) as losses of past ways of being in the world, embodiment, spatiality, and the future. In a systematic review by Hallberg (2004) into older people’s views on death and dying, older people showed a need to talk about existential issues, of time past and time to come, as well as of dying and death. If existential thoughts and reflections at this point are not recognized or affirmed, anxiety or existential loneliness (feeling alone in the world despite having people around) can arise (Sand & Strang, 2006). This suggests a need for further exploration to deepen our understanding of existential loneliness in the caring context.

Caring is an interpersonal interaction that is part of being human (Finfgeld-Connet, 2008; McCormack & McCance, 2010), and the professional’s main responsibility in encounters with patients and their families is to be caring. However, such caring may be constrained by nurses’ own insecurities and perceptions of older people’s attitudes towards death; nurses might avoid discussing existential issues and death in an effort to avoid reinforcing a patient’s feeling of hopelessness (Udo, Danielson, & Melin-Johansson, 2012). A study by Norell Pejner, Ziegert, and Kihlgren (2012) showed that although nurses considered giving emotional support to older people important, and knowing when it is needed as part of their professional skill set, work conditions did not always allow time for that. Ericson-Lidman, Norberg, Persson, and Strandberg (2013) showed that health care personnel were troubled by conscience when caught between various demands, rules, and recommendations that did not benefit the older people; they felt unable to relieve suffering and provide proper care to their patients. This indicates that being unable to work and act according to one’s values could create a feeling of guilt among professionals and promote neither good encounters nor good relations.

Another challenge for nurses was shifting their perspective from that of a busy professional in a care unit full of activity to the slower pace and “insiderness” of the older people’s perspectives. Although we can never fully understand someone else’s life world, “reaching towards” otherness as a process and practice is often more important than “knowing” the details of someone’s “insiderness” and is something to strive for (Todres, Galvin, & Dahlberg, 2014). Existential issues and caring are closely connected, and caring can be seen as an ethical aspect and a wisdom-based side of nursing (Udo, 2014). Thus, knowledge about nurses’ and other health care professionals’ encounters with older people perceived to be experiencing existential loneliness is vitally important to the development of supportive interventions for health care professionals.

## Aim

The aim of this study was to explore health care professionals’ experiences of their encounters with older people they perceive to experience existential loneliness.

## Method

This study forms part of the larger LONE study (Edberg & Bolmsjö, 2017) exploring existential loneliness among frail older people from the differing perspectives of the older people, their relatives, and their health care professionals. Frail older people were defined as those aged ≥75 years receiving long-term care from formal caregivers provided by the municipality or the county council. The LONE study is in the development phase of designing a complex intervention (MRC, 2008).

### Design

**Figure 1.**
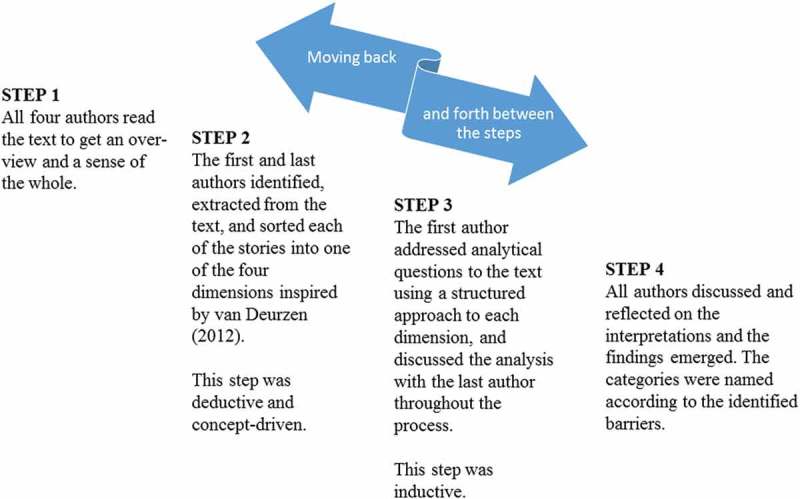
The four steps of the analysis.

This qualitative study was based on focus group interviews with professionals in different health care contexts. Focus groups were chosen to obtain a range of experiences (Krueger & Casey, 2009). We used a deductive–inductive approach in the analysis. According to Polit and Beck (2012), an emerging design based on reflective decisions can be used during the process of a qualitative study. In a first deductive phase, we used a concept-driven strategy (Schreier, 2012) based on a theoretical framework by van Deurzen (2012) followed by an inductive phase combined with an interpretative approach (Figure 1).

### Participants and context

In total, 11 focus group (FG) interviews were conducted in health care settings in urban and rural areas in the south of Sweden. For variation, we included home care, nursing home care, palliative care, primary care, hospital care, and pre-hospital care settings. Participants (n = 61) were also selected purposively to gain a range of caring perspectives from nurse assistants (licensed practical nurses and nurse aides), registered nurses, physicians, occupational therapists, physiotherapists, social counsellors, and social workers (Table I). Although the participants had different functions and tasks in relation to the older people, in this study are all referred to as *professionals* or *health care professionals*.

**Table I.** Characteristics of focus group participants.

Characteristics	n = 61	Specialist training
Age		
Range (md)	26–68 (49)	
Gender		
Women (%)	55 (90)	
Men (%)	6 (10)	
Profession		
Nurse assistant	22	
Registered nurse	25	
Physician	5	
Occupational therapist	2	
Physiotherapist	3	
Social counsellor	3	
Social worker/Case officer	1	
Additional specialist training/specialized orientation (˃ one year)	27	
Nurse assistant	3	Palliative care, Home care rehabilitation
Registered nurse	17	Oncology and palliative care, Primary health care, Pre-hospital care, Intensive care, Anaesthesia care, Elderly care
Physician	5	Oncology and palliative medicine, General Practitioner, Geriatrics
Occupational therapist	–	
Physiotherapist	–	
Social counsellor	2	Cognitive behavioural therapy, Psychodynamic therapy
Social worker	–	
Professional work experience in health care, *yr*		
Range (md)	4–43 (19)	
Work experience in the present organization, *yr*		
Range (md)	0.5–42 (9)	
Participants from each care context		
Home care	16	
Nursing home	11	
Palliative care	16	
Primary care	4	
Hospital care	9	
Pre-hospital care	5	

### Data collection

All FGs (n = 11) were conducted at the participants’ workplace in mixed groups of 3 to 8 professionals (md = 6). Prior to the interviews, participants were given written and oral information about the purpose of the study and assurance that their participation was voluntary and they could withdraw without any explanation. The information letter also included a brief text about existential loneliness in the context of caring and the LONE study as a whole. To safeguard the principle of autonomy, participants were given time between the first information and the FG to consider participating (Beauchamp & Childress, 2013). The information was repeated before written consent was collected and again before the interview.

The interviews were led by two researchers, one (M.S.) acting as facilitator and one (K.B. or M.R.) as observer. The interview guide was first tested in a pilot interview that led to a few adjustments. The interview guide began with an introduction. To focus the discussion, participants were then asked about their views on old people’s loneliness in general and existential loneliness in particular (Krueger & Casey, 2009). We requested stories about encountering existential loneliness by asking:

We are particularly interested in a deeper feeling of being alone in life, sometimes called existential loneliness, a feeling that can come and go. Can you remember an experience with an older person who had this deeper kind of loneliness, of being alone in the world? Could you please provide a concrete narrative?

Follow-up questions were asked when clarification was needed. The facilitator tried to create a supportive atmosphere to encourage interaction and reflection on the topic and emphazied that there were no right or wrong answers. The observer made notes and summarized the discussions at the end of the interviews, which lasted for approximately two hours and were audio-recorded and transcribed verbatim by a trained transcriber. One recording was interrupted by technical problems and the missing part reconstructed from the notes, augmented by an audio-recorded conversation between the observer and the facilitator shortly afterwards. The FGs were conducted from January 2015 to September 2016. Parts of the interview data that concerned existential loneliness in relation to the care context and strategies to handle existential loneliness will be presented elsewhere.

### Ethical aspects

One or two researchers from the LONE study informed participants about the project as a whole and handed out written information at meetings in the participating health care settings. For practical reasons, a designated contact person in each care setting then collected the names of those who wanted to participate and gave them to the researchers. The contact person was either a staff member or a manager, so there is a risk that some participants felt pressured to participate. However, the researchers highlighted the voluntariness of participation before each FG. The composition of the groups of staff from different professions in the same workplace could have influenced the interactions by making participants feel either comfortable or uncomfortable in the discussions. The researchers were aware of this possibility and tried to facilitate everyone’s opportunity to have their say without feeling constrained. This study was approved by the Ethical Review Board, Lund, ref. 2014/652, as a part of the LONE study.

### Pre-understandings

All authors are registered nurses with experience in geriatric nursing, education, and research. Our pre-understandings were articulated during the planning of this study. We defined existential loneliness as a feeling that could come and go among people in general. We assumed that existential loneliness is common among older people in need of care and that it is related to thoughts of death. We also believed that encountering older people’s existential loneliness was a challenge for which professionals could need support to meet.

### Analysis

The analysis was conducted in a process of moving back and forth between four steps (Figure 1). In the first step, all authors read the text to gain an overview and a sense of the whole. We looked for different aspects of health care professionals’ encounters with existential loneliness among older people and identified stories and descriptions related to the aim. In the second step, we structured these stories and descriptions on an analytical grid inspired by the life world theory of philosopher and existential psychotherapist, Emmy van Deurzen. Van Deurzen’s framework includes four dimensions: the *physical world*, the *social world*, the *personal world*, and the *spiritual world* (van Deurzen, 2012) (Table II).

**Table II.** Description of the four dimensions of van Deurzen’s theory of the life world (van Deurzen, 2012).

The Physical World	The most fundamental world, based on the assumption that human existence is rooted in the body, and includes the relation to nature, body, and oneself on a physical level.
The Social World	The world about the human existence in relation to others in the world with all aspects of social interaction as in ordinary meetings with others, human relations in the public world, and an inevitable part of life.
The Personal World	The world of closeness, to oneself and in other close relations. A psychological dimension including private experiences and identity.
The Spiritual World	The world about what creates meaning, about the person’s connection with the abstract elements in life and ideal values. About spiritual thoughts, beliefs and aspirations about life and the world beyond the person.

This step was concept-driven (Schreier, 2012) which is a deductive course of action using an already existent theory, concept, or, as in this study, a framework, where the life world theory acted as a coding frame. In the third step, analytical questions were addressed to the text:*What competencies and abilities are professionals using in encountering and interpreting older people’s existential loneliness? What are the professionals’ perceptions and interpretation of older persons’ existential loneliness? How do professionals describe that existential loneliness is expressed? What are professionals own experiences of encountering older persons’ existential loneliness?* In the fourth step, we looked for significant patterns in the answers to the analytical questions and categories to describe the challenges professionals faced in the encounters with older people’s existential loneliness. The overarching category was barriers in the encounter.

## Findings

**Figure 2.**
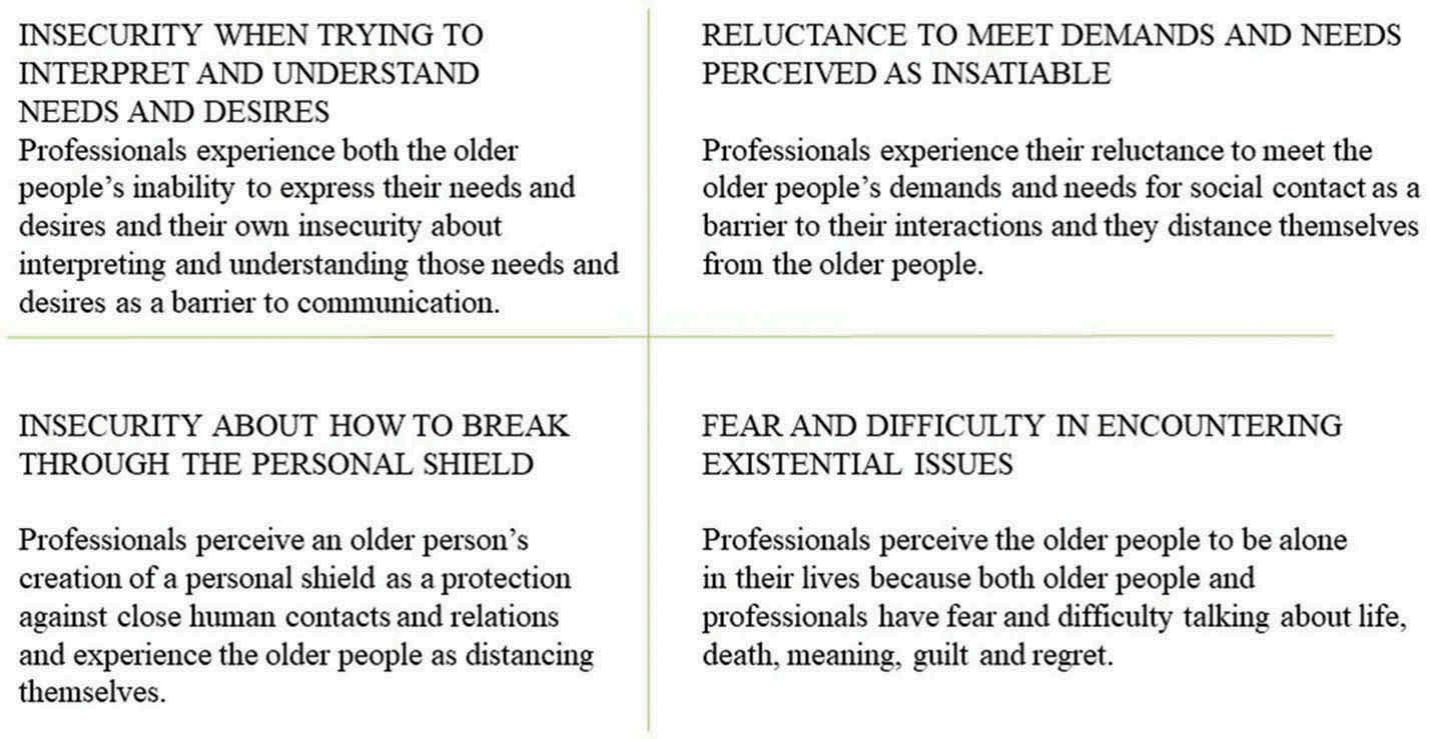
Professionals’ experiences of barriers in the encounter.

The professionals experienced existential loneliness among older people in various ways and situations. The condition was not static, but instead came and went. The professionals’ perceptions and interpretations of older patients’ existential loneliness and their own experiences of encountering existential loneliness are presented as barriers in the encounters: (1) *Insecurity when trying to interpret and understand needs and desires*; (2) *Reluctance to meet demands and needs perceived as insatiable*; (3) *Insecurity about how to break through the personal shield*; and (4) *Fear and difficulty in encountering existential issues* (Figure 2).

Regardless of what the barriers were about, staff described *individual characteristics and abilities* that made it easier for them to overcome these barriers. However, some characteristics and abilites were more prominent in some encounters. Characteristics such as empathy, compassion, courage, curiousity, and open-mindedness were helpful in overcoming barriers, and the abilities to listen, to empathize, to reflect, and to switch perspective to the older person’s life world seemed significant in the encounters. Familiarity with the person’s past and present history, culture, and society were also important for professionals to comprehend the older people’s situations. Finally, professionals’ own norms and preferences combined with lack of knowledge of the ageing process could hinder professionals’ abilities in encounters with existential loneliness.

### Insecurity when trying to interpret and understand needs and desires

The professionals experienced insecurity when bodily limitations due to, among others, cognitive impairment, impaired hearing, and severe pain obstructed communication and interaction with others. They interpreted that the feeling of existential loneliness was characterized as being sad, abandoned, feeling anxiety, feeling fear, and being vulnerable and isolated.

Losing the ability to express themselves due to dementia or frailties limited older people’s ability to express needs and desires. Cognitive impairment was a barrier that led to difficulties for older people in understanding what was happening and influencing their existence. A situation like that could be when an older person with dementia was separated from relatives when being a resident in a nursing home:

Yes, but I’m thinking about … those times when they [her significant others] are not there, and she is still so sad, and she cries and cries, then she is sad because she is aware that, well “I’m here and they’re there”, and she misses them and longs for them. (Interview 7—story 6)

To have needs, but be prevented by bodily frailty from expressing oneself or being understood by others led, according to the professionals, to isolation and existential loneliness. Even older people who prefer to withdraw to avoid misunderstandings, such as patients with a hearing impairment, were perceived to be isolated and separate from others. Professionals interpreted existential loneliness among older people with bodily limitations as an inability to escape, being condemned to see the world but not be a part of it, or as living in a world of their own.

Professionals described different expressions and signs of existential loneliness among older patients. Seeking contact by calling for attention in vulnerable situations was considered a sign of existential loneliness:

When they’re lying there, crawling, or knocking with their cane on the wall, or knowing that the newspaper carrier is coming at five in the morning or something like that, that’s when I can call for help. It feels like there’s a deep feeling of loneliness in all that. (Interview 3—story 1)

Professionals’ encounters with older patients’ existential loneliness made them feel insecure, inadequate, and powerless due to the barrier of bodily limitations, but, in some cases, also compassionate. Feelings of inadequacy arose from not being able to reach the older people or interpret their needs and desires and instead having to rely on guesswork. Some professionals also experienced anger as a sign which was difficult to understand and get through. Encounters marked by mutual understanding were considered significant moments, especially when there were communication problems, such as in this case of dementia in a patient with Swedish as a foreign language:

“Oh yes, it is absolutely amazing those days when she’s spending time with you and she’s talking a few sentences in Swedish [and you can connect]” (Interview 7—story 6).

Older people’s loss of independence evoked compassion and sadness in professionals, sometimes because of similar experiences with their own relatives. This helped professionals to open up to reflections about the conditions of life that could be used in their work with older people.

### Reluctance to meet demands and needs perceived as insatiable

The professionals’ experiences of older people’s demands and needs for social contact with professionals or family members sometimes provoked in them a form of reluctance that acted as a barrier to interaction. Professionals considered older people’s claims to, and longing for, contact as expressions of an existential loneliness characterized by sadness, homesickness or nostalgia, anxiety, anger, and fear of death or of dying alone. They also reflected that existential loneliness in older people was connected to feelings of invisibility and being forgotten.

Professionals’ experience of older people’s demands and needs for compassion made the professionals wish to distance themselves. Some of the patients had a strong need and desire for one person who could not be replaced. No amount or type of contact from professionals was ever enough:

“But even though you [home care staff] are there, there is still this empty space. Even though you are the one who is going there every day” (Interview 8—story 7).

Hospital discharge was seen as a loss of safety for older patients for whom the hospital represented a sense of security. Vulnerability and loneliness at home were interpreted as threats to older people that could cause them severe pain or prompt them to call for an ambulance after discharge. Such frequent and recurrent help-seeking was also interpreted as a sign of difficulty in managing anxiety and loneliness in other ways. The professionals understood the patients’ attempts to reach out for human contact and their longing for closeness and intimacy in the context of demands and needs perceived as insatiable as an expression of existential loneliness. The need for companionship was perceived to get stronger in times of loss and uncertainty.

The professionals described expressions and signs of existential loneliness differently. Older people using jargon or casual language could be a disguise for existential loneliness. Professionals considered that although older people’s needs for companionship and attention from professionals remained constant, how older people behaved towards professionals and others differed. Another sign was when older people looked lonely even when they were with other people. Professionals also noticed more existential loneliness in older people who rarely had visits. These persons took an active approach to seeking contact, attention, and confirmation from professionals; some sought physical contact and others tried to prolong the conversation:

I recognize this, that is sort of being dragged into the space around these patients: “Don’t go … ”, and the [older] person tries to keep the conversation going and all that, to make you [health care professional] stay around for a bit longer. Or [the older person] talks very, very slowly so that the conversation goes on for a very, very long time. (Interview 6—story 2)

Professionals’ encounters with their patient’s existential loneliness were characterized by feelings of frustration and stress. They felt inadequate when they could not satify the older person’s needs no matter how much they did:

“Yes, oh yes, it’s all the time, talking and talking, and it’s like … you are there for socializing [an assessed activity] for an hour and you do things together, but still you still feel you haven’t done enough” *(*Interview 6—story 3).

This could leave professionals feeling drained by relations that required both time and energy and were described as being dragged into a situation that was difficult to escape. The professionals felt they lacked suffient time and resources to support the older people properly, especially those who expressed or repeated their wish to die. Professionals hesitated about when it was time to discuss existential issues. During good encounters, their insight and understanding of the older person’s situation grew and the relation became less enervating.

### Insecurity about how to break through the personal shield

Professionals experienced insecurity when older people were perceived to distance themselves through a sort of psychic personal shield used as a protection against close human contacts and relations. This shield was understood to represent the person’s reluctance to let anyone share their private sphere and it acted as a barrier to the relation between the professional and the older person. Professionals associated this shield with people who had always lived by their own rules and relied on themselves. They interpreted the existential loneliness of these people, characterized by anxiety, bitterness, and anger, as a result of their inability to maintain their own rules and independence. It was a painful feeling, deep inside the older person, that the professionals sensed as overwhelming.

Professionals experienced ambivalence in older people towards letting go of control and letting others, including family members, get close. These people had no one to talk intimately with and were perceived to keep their problems and existential thoughts to themselves. They were understood not to allow themselves to be weak, vulnerable, or dependent. These people also seemed unable to verbalize or communicate their situation and existential issues:

“so suddenly there comes a loneliness that you may not be able to communicate to others. And you may not even be able to put your finger on it yourself, either. It’s just something that comes over you” (Interview 5—story 1).

The pleasant loneliness of solitude and the private sphere that was once a sanctuary no longer has the same value when family members, especially children and grandchildren, are occupied with their own lives. Although some older people said that their relatives were too busy to visit, they often talked *about* their relatives without really knowing (as the professionals often did) about their lives, their concerns, or their wishes to know more about the older person’s situation. Another sign of existential loneliness, then, was when older people expressed loneliness while at the same time declining company:

She sometimes expresses feelings of loneliness, but when you go into her room and suggest something to do, she says “No, I don’t want to cause any trouble to anyone …” There is that kind of people, too. I don’t know … they feel that they don’t want to put any burden on you … and so on. So it can be like that, too, that some people think “No I don’t want to talk … or not talk …”, like “I don’t want to be a problem to anyone.” (Interview 8—story 6)

Professionals saw older people having difficulty in starting new relationships after the loss of relations and changes to their circumstances. When a professional found a “way in”, and broke through the personal shield, the older person might express gratitude later after having resisted. Professionals identified a passive attitude in some older people who took very few initiatives and chose to stay behind the shield. However, others had a genuine need for peace and quiet before death and some wished to die alone in privacy.

Professionals’ own encounters with existential loneliness were characterized by their commitment and will to get behind the older person’s shield, but they felt insecure about how to do this. They expressed feeling responsibility for supporting the older people’s well-being and felt that their actions were crucial. However, when they remained locked outside and not admitted behind the shield, they felt they had not done enough, especially when they sensed that they could ease the patients’ feelings of existential loneliness through their support and understanding. They also tended to feel insecure when their own perceptions differed from what was customary according to the core values of their profession. One example was the rule not to allow anyone to die alone:

But she didn’t want [to have people around when she was dying] and the children did not want [to go against the will of their mother]. There were a lot of discussions around here; it aroused many … different kinds of feelings, because I think we put ourselves into her situation or into the children’s situation. Like, “What is this? How would I feel if I were lying there?” Thats the way you think. But on the other hand, it’s not me. (Interview 11—story 3)

When older people expressed strong emotions, professionals often perceived them as a wall that hindered their ability to get close to the older person. This awoke their compassion and was remembered for years as an object of reflection and learning. When the patients chose to trust them and allowed them to break through the shield, professionals felt a sense of happy amazement.

### Fear and difficulty in encountering existential issues

The professionals experienced fear and difficulty in encountering existential loneliness among the older people they perceived as existential lonely. At these moments, when encountering existential issues and aspects of life, they sensed anxiety, agony, aimlessness, and disappointment in the older people. These emotions were understood as feelings of rootlessness, abandonment, and hopelessness in the present situation. Most of these concerned the past, but older people also expressed concerns about the meaning of their present lives and futures.

Professionals also perceived that older people were often occupied by brooding reflections on their lives, imminent deaths, feelings of guilt, and regrets:

But this, I perceive as loneliness. His wife didn’t understand him, we didn’t understand him, and he didn’t understand himself either. And it is about life and death then … and he … he was all into that now and he had regrets. (Interview 5—story 5)

Some older people were able to express their existential loneliness in words, but others did not know how. Conversations about existential issues and loneliness were sometimes intertwined with other topics. Expressions of regret and guilt over broken relationships or choices in life were interpreted as signs of existential loneliness, as were expressions of feeling useless, no longer significant to others, and alieniated from contemporary society. The professionals also noticed older patients’ longing for people from their own generation who had experienced the same events and episodes they had. Others were focused on their body and signs of weakness and decline. Smoothing things over, apologizing, and hinting about feelings of shame were other signs of existential loneliness. Although professionals sensed these signs could be caused by existential ruminations, their own fear and difficulty in talking about existential matters were barriers in the encounter.

Professionals’ encounters with older people’s existential loneliness aroused existential concerns within themselves. The existential loneliness was considered to affect everyone involved and was difficult to endure, particularly when it was the professionals’ own inability and fear that created the barrier. Reflections brought new insights about the encounters and the complexity of existential loneliness. Professionals were aware of their limited ability to eliminate the feeling of existential loneliness. Another insight was the importance of letting go of their own personal views of the situation. Talking about existential issues with older people required self-awareness and courage:

To me, being is existential. Not so much about religion, it is about living. Just to live your life, being here and now, to exist in full, to be whole. That is existence, I want us to dare to talk more openly about it. (Interview 10—story 3)

Another reflection the professionals expressed was the importance of prioritizing conversation and talking about death and dying before it was too late. The opportunity to share existential thoughts with an older person brought them happiness and gratitude, and these encounters were remembered for years.

## Discussion

**Figure 3.**
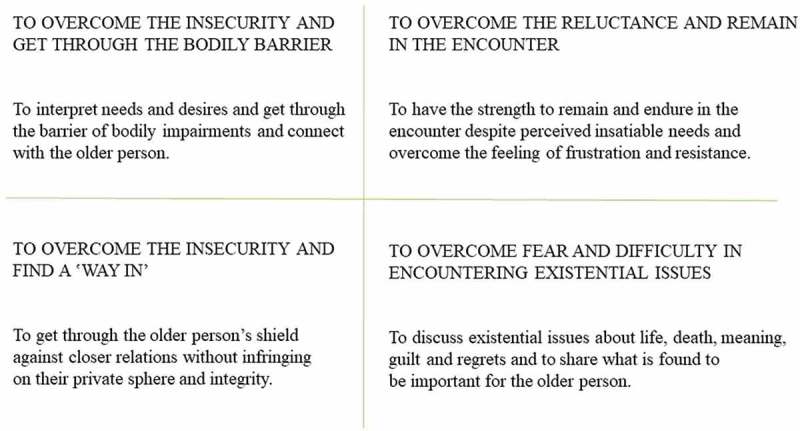
Challenges in encountering older people’s existential loneliness.

The results pointed out the different barriers faced by professionals encountering existential loneliness in a caring context. These encounters with existential loneliness affected health care professionals’ feelings of various kinds and to various degrees. The four identified barriers in the encounters, *insecurity when trying to interpret and understand needs and desires, reluctance to meet demands and needs perceived as insatiable*; *insecurity about how to break through the personal shield*; and *fear and difficulty to encounter existential issues* demand different approaches. This means facing different challenges of requiring different personal qualities such as empathy, courage, curiousity, and open-mindness in the encounter (Figure 3).

Obstructed communication and interaction due to cognitive impairment and bodily limitations was perceived and interpreted as giving rise to isolation and existential loneliness. However, the results showed that interpretations of the older people’s experiences often had to be based on guesswork. We also found that professionals sometimes felt inadequate and insecure about how to connect to older people; for example, when an older person, because of impaired cognitive function, could not fully understand or grasp the situation. The challenge for professionals was therefore to overcome a barrier in terms of their own *insecurity to interpret and understand these people’s needs and desires*. To connect to the older person requires empathy and the ability to enter into the other’s life world. Professionals need to support older people, especially those with impairments, to maintain a caring encounter and avert possible existential loneliness, as highlighted in a study by Nyström (2006) of people with aphasia who suffered existential loneliness when unable to express themselves. These people also felt frustration and anger in their struggle to communicate, and existential loneliness in the absence of professional support. In a case study on aphasia, communication loss was a hindrance to self-expression as a thinking and acting person that led to feelings of exclusion and changed the process of natural conversation in a world of others (Hjelmblink, Bernsten, Uvhagen, Kunkel, & Holmström, 2007). Being unable to share thoughts left the person feeling alone in the world. To improve the quality of elder care, the impact of physical constraints on older people’s feelings of alienation and loneliness must be brought into light along with the effect of professionals’ feelings of inadequacy.

Older people’s claims to and longing for social contact was perceived and interpreted as expressions of an existential loneliness that made the professionals distance themselves. Our findings showed that professionals often felt frustrated and inadequate. Having the support of their colleagues and opportunities to discuss and become aware of their own behaviours could help professionals find ways to deal with the situation. The challenge to overcome the *reluctance to meet demands and needs perceived as insatiable* requires the professional to have the strength to endure the encounter. The professional needs to meet the older person’s feelings without becoming overwhelmed or exhausted. According to Charmaz (1983), people with chronic illnesses are still in great need of intimate social contacts, but can have difficulty maintaining relations because they are consumed by their illness and circumstances and therefore become more and more isolated. This could be one reason professionals distance themselves from patients. However, distancing may also be a way that professionals handle demanding situations and it should therefore not be overlooked as a potential sign for colleagues or managers to pay attention to. Group supervision is one alternative that, apart from offering an opportunity to share demanding situations with colleagues, could be an opportunity to simplify complexity and increase professionals’ self-awareness and confidence (Taylor, 2014). Buber (1994) claimed that the person, in this case the professional, who knows the difference between “I and thou” and “I and it” can switch between the two to find meaning. However, if a professional is caught in one of those relations to the world, it can lead to consequences for both parties in caring and non-caring encounters. Reflecting with colleagues on the relationship between existential loneliness and health care could open up discussions of alternative approaches. Westin and Danielson (2007) showed that encounters with nursing home staff influenced older people’s feelings of “being somebody” or “being nobody”. Both residents and professionals effected the encounters, although professionals were responsible for the outcomes. An encounter that makes an older person feel like a “nobody” or an “it” is not caring and should be prevented, or acknowledged and remedied.

The older people’s distancing due to a reluctance to share their private sphere was perceived and interpreted as giving rise to existential loneliness and painful feelings. Our results showed that professionals were committed and felt responsibility for supporting the older people but felt unsatisfied when being locked out. The challenge of overcoming the *insecurity about how to break through the personal shield* is twofold. It is necessary first to find a “way in” and second to do so without infringing on the older person’s integrity. Because professionals were found to associate the personal shield with a person’s character, it is important they have some knowledge of the person’s personality and life story before the encounter. The older person may have a history and values of strength and independence that are important to respect. Tillich (1963) described a person with an impenetrable centre as free and this freedom is an aspect of being alone. This could be interpreted as integrity, but when being alone is no longer self-chosen and circumstances change, the glory of such solitude can fade. Professionals interpreted existential loneliness as a painful feeling, connected to the person’s very existence, which implies that it can be appropriate to “knock on the door”, but it is important not to be overconfident or insistent. Instead, a combination of courage and attention to the person’s possible vulnerability is part of moral strength on the relational level (Lindh, Severinsson, & Berg, 2009). This could open patients to encounters on their own terms. Nevertheless, it is important to respect the older person’s integrity and not misinterpret self-choosen solitude as loneliness.

Older people being occupied by deep reflections on their lives in relation to guilt, regrets, and their current situation, but missing having anyone to share existential issues with, were interpreted as experiencing existential loneliness. Professionals experienced this kind of existential loneliness as difficult to endure, in particular when the professionals’ own inability and fear hindered them. The results showed that professionals identified older people who were able to express their existential loneliness and distress in words, while others either did not know how or disguised their existential loneliness with other words or expressions. The challenge of overcoming *fear and difficulty in encountering existential issues* is to challenge one’s own insecurity and try to find ways to share the older person’s existential distress. Professionals therefore need to be attentive to signs of existential distress and open to discussions without feeling obliged to have the answers to everything. Frankl (2006) believed the meaning of life has to be found by each individual and is in constant, unceasing change, including during ageing and at the end of life, when reflection and brooding over existential issues are common. This is crucial and should not be underestimated or overlooked in ageing people, especially as there is a risk that assumptions and preconceptions about older people in contemporary society can lead to neglect of their needs. A study of health care professionals’ perceptions found that among patients with advanced cancer, anticipation of a negative future, failure to find meaningful activities and relationships, and feelings of regret were causes of existential distress (Mok et al., 2010). Frankl (2006) emphasized the potential of life, even of the past, and emphasized that the accomplishments, joys, and sufferings of the past all give meaning to the present and the future. This strengthens the importance of cultivating an interest in the older patient as a person throughout time. When the barrier is the professionals’ own fear, they first have to overcome that fear and develop courage and self-awareness to encounter whatever existential issues older people will share with them. One way of developing courage and self-awareness is through clinical supervision based on reflection in dialogue with others. Core concepts, besides self-awareness, in the nursing supervision model are confirmation and meaning (Severinsson, 2001). Self-awareness as well as awareness of the importance of encountering existential issues can most probably improve the care provided. Schuster (2006) pointed out the importance of becoming aware of the joint experience of being human, but also highlighted the importance of confirming the differences between the unique patient and the professional. The meaning of being professional is often discussed in the sense that one should not become personal in relation to the patient. Schuster (2006), however, denoted that in a professional existential space, the private sphere is left behind, and she claimed the possibility of being personal and still professional. To reach this, courage is required.

Depending on how professionals deal with the barrier, the encounter will become caring or uncaring. Caring encounters, as defined by patients, are provided by competent, concerned, respectful, open, and positive professionals who give patients an increased sense of well-being (Halldórsdóttir & Hamrin, 1997). Our results are similar, showing that professionals’ compassion, open-mindedness, and humility towards people’s differences could serve as threefold basis for caring encounters and meeting existential loneliness. To create caring encounters and give older patients a sense of well-being, it is important for professionals to have opportunities to reflect on the care they give and on the existential issues their patients face. Ranheim (2009) stressed not only the importance of enough time in encounters with patients to recognize their existence as individuals, but also the need for professionals with enough sense to answer the “silent call” and the self-awareness and strength to recognize underlying concerns as well as explicit and easily solved issues. Existential issues concern all human beings—patients, older people, health care professionals, and everyone else. It is important to recognize and acknowledge the significance and meaning of existential issues in the caring relationship to provide good-quality care and support older persons’ well-being.

### Methodological considerations and limitations

Because there is no clear consensus on the concept of existential loneliness, we did not choose one definition. This may be seen as a weakness and a challenge to the credibility of our data (Guba, 1981). To counter this possibiliy, we introduced the concept to participants in the written information and introduced all the focus groups to the description of existential loneliness as a deeper kind of loneliness, a feeling of being alone in the world. The interview guide was first tested in a pilot interview with five people. They were all nurses, either students at advanced level or lecturers in a nursing programme. The pilot interview led to some adjustments of the guide and made us aware of the importance of requesting stories. Therefore, we asked for stories, which we expected would lead to depth in the interview data; however, we noticed a difference between participants in the degrees of their personal and emotional involvement and reflection in the stories. One interpretation could be that the professionals had experienced and reflected about existential loneliness to varying degrees; another could be that the climate in some focus groups hindered the participants from becoming very personal. Still, we believe that most focus groups had an open climate, although this did vary with different group dynamics. Further, the professionals did share many stories, sometimes from years ago, that could be interpreted as significant memories to them on both a professional and a personal level. The number of professionals in the groups varied between three and eight, which might have affected the opportunity to speak. Still, our opinion is that group dynamics and to let everyone have their say were more important for the climate than the number of participants. The study design involved participants from different professions and care contexts to help us achieve a result that could be transferable to encounters with older people in various care contexts. However, the participants in our study were probably more engaged in existential issues than professionals in general. If so, insecurity about encountering existential loneliness might be even more widespread.

The deductive–inductive design emerged over time, and according to Guba (1981) a naturalistic inquiry is not complete until it is terminated. During that time it is unfolding and not preordained. Using van Deurzen’s framework (2012) was beneficial in explaining and understanding other aspects of existential loneliness, such as the frail body, and relations to oneself and to others. We described all four steps of the analysis and reported the analytical questions to increase dependability by establishing an audit trail (Guba, 1981). All four authors took part in various parts of the analysis over time including data collection, analysis, and drafting this paper, which strengthens the dependability and the confirmability of the study. We strived not only for confirmation of our hypotheses, but also to challenge our pre-understandings and see the interpretations in a new light. Our pre-understanding was initially that existential loneliness would to some extent concern dying and the time before death. However, the results surprised us somewhat by showing the experience of existential loneliness in a wider perspective, as well as the barriers experienced by professionals. We were thus made aware of our initial prejudice and we were able to show our willingness to expand the horizon of meaning (Gadamer, Weinsheimer, & Marshall, 2004; Nyström & Dahlberg, 2001). In addition, the results have been presented and discussed in the research group as well as a reference group, both connected to the LONE study. The reference group has met twice during this time and the members have various experiences from health care, as a professional, informal carer, or relative.

## Conclusions

Encountering existential loneliness is experienced by health care professionals as both challenging and meaningful, and it is important to talk about it and highlight its role in providing good-quality care to older people. It is demanding for health care professionals to encounter existential loneliness and to discuss issues such as the meaning of life, death, guilt, and regret, which prompt professionals to reflect upon existential aspects of their own lives. Consequently, the effects of facing existential loneliness do not seem to be limited to the caring situation, but rather to affect health care professionals both personally and professionally. Giving health care professionals time to reflect, both with their colleagues and alone, could increase their self-awareness and significantly improve the quality of care for older people in the later phases of their lives.
